# Good clinical activity and favorable toxicity profile of once weekly bortezomib, fotemustine, and dexamethasone (B-MuD) for the treatment of relapsed multiple myeloma

**DOI:** 10.1002/ajh.23358

**Published:** 2012-12-08

**Authors:** Silvia Mangiacavalli, Lara Pochintesta, Cristiana Pascutto, Federica Cocito, Mario Cazzola, Alessandro Corso

**Affiliations:** Division of Hematology, Fondazione IRCCS Policlinico San Matteo, University of PaviaPavia, Italy

## Abstract

Since multiple myeloma (MM) is still not-curable, the management of relapse remains challenging. Given the known efficacy of alkylating agents in MM, we conducted a phase I/II study to test a new three drug combination in which Fotemustine (Muphoran), an alkylating agent of nitrosurea family, was added to bortezomib + dexamethasone backbone (B-MuD) for the treatment of MM relapsed patients. Fotemustine was administered at two dose levels (80–100 mg/m^2^ i.v.) on day 1. The original 21-day schedule was early amended for extra-hematological toxicity and a 35-day schedule was adopted (Bortezomib 1.3 mg/m^2^ i.v. on days 1, 8, 15, and 22, Dexamethasone 20 mg i.v. on days 1, 8, 15, and 22) for a total of six courses. Twenty-four patients were enrolled. The maximum tolerated dose of Fotemustine was 100 mg/m^2^. The overall response rate was of 62% (CR 8%, VGPR 33%, and PR 21%). The median OS was 28.5 months, the median progression-free survival (PFS) was 19.1 months. B-MuD resulted effective in patients previous exposed to bortezomib without difference of response (*P* = 0.25) and PFS (*P* = 0.87) when compared to bortezomib-naive patients. Thrombocytopenia was the most common AE overall. In conclusion, B-MuD is an effective and well tolerated combination in relapsed MM patients even in advanced disease phase. © Am. J. Hematol., 88:102–106, 2013. © 2012 Wiley Periodicals, Inc.

## Introduction

Multiple myeloma (MM) is highly sensitive to alkylating agent, particularly to melphalan; oral low-dose melphalan in elderly patients and high dose melphalan for the younger ones still represent the backbone of the treatment of MM. Although the introduction of novel agent (Thalidomide, Lenalidomide, and Bortezomib) has significantly changed the scenario of MM 1 nearly all patients relapse and ultimately develop a refractory disease 2,3. Thus, it is still important to identify new compounds active against the plasma cell clone. Fotemustine, a cytotoxic alkylating agent belonging to the nitrosureas family, recently used in an alternative condition regimen (fotemustine plus etoposide, cytarabine, and melphalan) for lymphoma patients 4, has proven to be active when used as single agent in the MM relapsing setting. It is delivered by short course i.v. infusion not requiring hospitalization; given its pharmacokinetic characteristics there is no need for dose reduction in presence of altered renal function. Myelosuppression, and in particular thrombocytopenia, is the most common reported toxicity 5,6. Several studies have shown the synergic activity of bortezomib with the alkylating agent melphalan whether in combination with prednisone in elderly patients or with dexamethasone in relapsed patients, observing a final response rate of about 70%, with a rate of high quality response (≥VGPR) ranging from 15 to 34% 7–9. Considering the importance of achieving a high-quality response 10–14 even beyond frontline setting 15,16, and the good safety profile observed for single agent fotemustine, we conducted a dose escalation clinical study to evaluate the tolerability and the activity of a combination therapy including fotemustine, bortezomib, and dexamethasone.

## Methods

### Patients

MM patients with active progression after at least one line of therapy where eligible for the study. Patients who received prior bortezomib-containing regimen were included only if not considered bortezomib-refractory. Additional eligibility criteria included presence of measurable serum or urine paraprotein, a Easter Cooperative Oncology Group performance status of <3, platelet count >100·10^9^/l, absolute neutrophil count >1·10^9^/l, and serum creatinine <2 mg/dl, serum hepatic aminotransferase levels ≤3 the upper limit of normal or a total serum bilirubin ≤2 the upper limit of normal, or absence of other serious medical illness that could potentially interfere with the completion of treatment. Patients with peripheral neuropathy (PN) grade ≥2 or patients receiving any investigational drugs within 14 days of enrollment were excluded. The protocol was approved by the our Local Ethics Committee in accordance with the Declaration of Helsinki. Participants provided written informed consent prior to enrollment.

### Study design and drugs administration

The primary objectives of this monocentric, non-randomized, phase I/II dose-escalation study were to determine the maximum tolerated dose (MTD) of the fotemustine/bortezomib/dexamethasone combination (Phase I) and to determine the overall response rate (ORR) and the safety profile of the combination once established the MTD (Phase II). Secondary objectives were to assess overall survival time to progression (TTP), progression-free survival (PFS), time to first response, duration of response (DOR), and time to next treatment (TNT). Each of those latest variables were defined according to recent updated criteria for uniform reporting of clinical trial 17. In addition, we reported treatment free interval, defined as time from the latest dose of experimental therapy and the first dose of next therapy or time of last observation. Experimental therapy originally consisted of two escalating dose of fotemustine (80 or 100 mg/m^2^ i.v.) on day 1 of each 21 day-cycle. Patients received a fixed dose of Bortezomib 1.3 mg/m^2^ i.v. on days 1, 4, 8, 11, and Dexamethasone 20 mg orally on days 1–2, 4–5, 8–9, and 11–12. The two escalating doses were tested following the Bayesian method 18 and the calculation of the sample was made according to the model of Ji et al. 2007. Fotemustine dose was increased if six consecutive patients completed two cycles without a dose limiting toxicity (DLT). The MTD was defined as the highest dose level at which 30% or fewer patients experienced a DLT. DLT was defined as the occurrence of grade IV haematological toxicity or grade III non haematological toxicity. Since extra-hematological toxicity deemed unacceptable was encountered after the enrolment of first six patients, following our ethics board approval, the original schedule was amended. The modified schedule consisted of the same escalating dose of fotemustine (80 and 100 mg/m^2^ i.v.) plus once weekly Bortezomib 1.3 mg/m^2^ i.v. on days 1, 8, 15, 22 and Dexamethasone 20 mg i.v. on days 1, 8, 15, 22 of each cycles. Each cycle was then repeated every 35 days for a maximum of six cycle. As fotemustine and bortezomib clearance are independent of renal function, there was no need for dose reduction in patients with renal impairment. Bortezomib dose reduction (from 1.3 to 1 mg/m^2^ and then to 0.7 mg/m^2^) was applied in accordance to established guidelines 20,21 in patients experiencing grade ≥2 PN or any grade 3 or higher non-hematologic or grade 4 hematologic toxicity.

Entry in the next cycle was held for neutropenia or thrombocytopenia of ≤1·10^9^/l or ≤75·10^9^/l respectively or persistence of any extra-hematological adverse event of NCI grade ≥2; treatment was discontinued if the adverse event did not resolve within two weeks.

### Efficacy and safety measurements

Pretreatment evaluation consisted of patient history, physical examination, electrocardiogram, and chest radiographs. Neurologic examinations were conducted by the physician at screening and then at the beginning of each cycle, and at the end of study. Blood and urine samples were collected at screening and on days 1 of any cycle except for hematology which was performed before each bortezomib dose. After treatment completion, patients were monitored every 8 weeks until disease progression. A negative pregnancy test was required for all women of childbearing potential. Adverse events were graded according to National Cancer Institute Common Terminology Criteria for Adverse Events, version 3.0. (CTCAE) (http://ctep.cancer.gov/forms/CTCAE_Index.pdf). Responses were graded according to IMWG criteria 22,23 and patients were considered responsive when achieving at least a partial response (≥PR). Monitoring of response was performed after each treatment cycle and at study-end by means of quantification of serum immunoglobulins, serum protein electrophoresis, immunofixation and collection of 24-hr urine specimen for total protein, electrophoresis and immunofixation. Bone marrow plasmacytosis and skeletal radiological evaluation was included in response evaluation only if indicated. Patients were considered evaluable for response when completing at least 2 cycles.

### Statistical analysis

Numerical variables were summarized by median and range; categorical variables by count and relative frequency (%) of subjects in each category. Comparison of numerical variables between groups was carried out using a nonparametric approach (Mann–Whitney test). Comparison of the distribution of categorical variables in different groups was performed with the Fisher exact test. Survival analysis was carried out with the Kaplan–Meier method, and the Gehan–Wilcoxon test was applied to compare survival curves.

To assess the effect of response on disease progression, we performed a survival analysis defining the entry time as the date of response assessment. Patients who did not complete the study entered this analysis at the time of drop-out.

## Results

### Patients and dose escalation

A total of 24 patients were enrolled between May 2009 and March 2011. Patients characteristics were summarized in %Table 1. The median time from diagnosis to study entry was 64 months (range 14–155 months). Number of previous therapies were 2 (1–5). Previous treatments included autologous transplant in 13 pts (54%), bortezomib in 8 pts (33%), oral melphalan in 11 pts (46%) and thalidomide in 15 (63%). At the time of the analysis, all patients enrolled had completed the treatment schedule.

**Table 1:** Patients Characteristics at Study Entry

Characteristic	Patients (*n* = 24) %
Median age years (range)	69 years (44–83)
Gender *n* (%):	
• Male	13 (54%)
• Female	11 (46%)
Paraprotein Isotype *n* (%):	
IgG	14 (59%)
IgA	8 (32%)
Light chain only	2 (9%)
ISS stage^*^:	
I	6 (25%)
II	10 (42%)
III	8 (33%)
Chromosome abnormality:	
none	12 (50%)
del 13	4 (18%)
*t* (4;14)	2 (8%)
*t* (11;14)	2 (8%)
*t* (14;16)	1 (4%)
del 17	3 (12%)
Haemoglobin:	
Median (range) gr/dl	11.9 (9.8–15)
<10 gr/dl	4%
Platelet count:	
Median (range) 10^3^/mmc	174 (73–310)
<150 . 10^3^/mmc	25%
Serum Creatinine:	
Median (range) mg/dl	0.79 (0.5–1.73)
>1.5 mg/dl	4%
Serum Calcium:	
Median (range) mg/dl	9.7 (8.1–10.6)
>10 mg/dl	18%
Lactate dehydrogenase:	
Median (range) U.I./l	347 (207–633)
>400 U.I./l	37%
B_2_microglobulin:	
Median (range) mcg/l	3460 (1,840–7,220)
>2,500 mcg/l	88%
Therapies prior to study entry	
Median *N*° of regimen	2 (1–5)
Type:	
• Autologous transplant	13 (54%)
• Bortezomib	8 (33%)
• Oral melphalan	11 (46%)
• Thalidomide	15 (63%)
Time from diagnosis to study entry: Median (range) months	64 (14–155)

DLTs registered after the enrolment of the first cohort of six patients during the dose escalation phase of fotemustine, were as follows: three patients experienced PN of grade 3–4, one patient registered a grade 4 thrombocytopenia, one a grade 3 pneumonia. Because of those toxicities deemed unacceptable the schedule was amended as specified above. The dose escalation of fotemustine was completed after completing two consecutive cohorts of six patients treated according to the amended schedule; as a result the MTD for fotemustine was established to be 100 mg/m^2^. The median percentage of planned dose delivered was 93% (range 10–100%).

Six of out of 24 patients enrolled (25%) did not completed the assigned six cycles. Four pts discontinued for extra-hematological toxicity (one patient had grade 3 gastrointestinal bleeding after the first cycle, one grade three pneumonia after the second cycle, one grade 4 PN after the third cycle, one sepsis after fourth cycle), two for progression both after completing the fourth cycle.

### Efficacy

**Figure 1:**
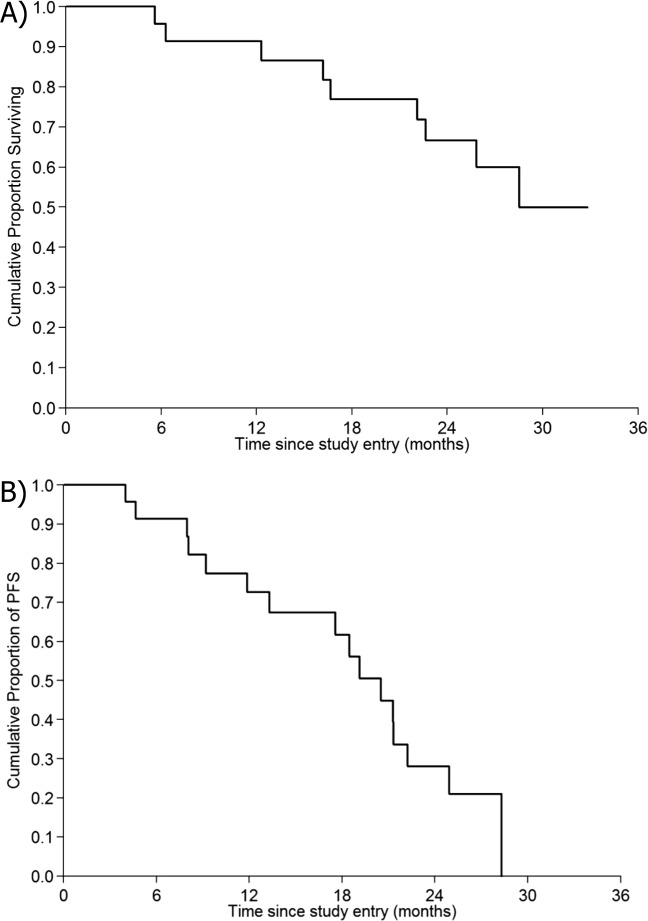
Outcome. Kaplan–Meier estimates of OR (A) and progression free survival (B) from study entry of 24 MM patients treated with B-MUD.

**Figure 2:**
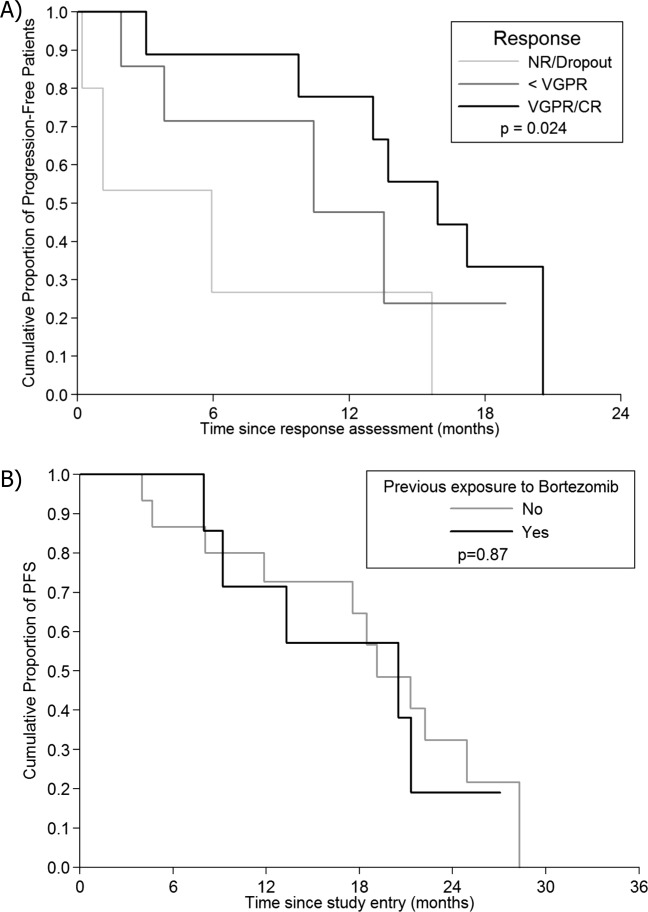
Progression free survival (intention to treat population) according to response (A) and previous exposure to bortezomib (B).

The efficacy analysis made on intention-to-treat-basis showed a final ORR (≥PR) of 62% (CR 8%, VGPR 33%, PR 21%) with 9% of SD, and 4% of patients with progression (%Table 2). Median time to first response was 36 days (range 21–83), half of patients reached their best response within the third cycle, the median DOR was 19.4 months (95% CI 11.6–23.7 months). The median duration of follow-up from study entry was 24.3 months (range 1.6–32.8 months). Progression or relapse occurred in seventeen (71%) of 24 patients, with nine patients (37%) died at the time of the analysis, all for progressive disease. The median OS is 28.5 months (95% CI 22.1-NR) (Fig. 1). The median TTP and the median PFS were 20.5 (95% CI 11.9–22.2 months) and 19.1 (95% CI 11.9–22.2) months respectively. The median TNT was 10.6 months (range 0–22.6 months). The median progression free survival in patients who received bortezomib + dexamethasone backbone (B-MUD) as second or third line therapy was 21.3 months as compared with 13.3 months in patients treated in more advance phase, thought this difference was not statistically different (*P* = 0.43). There was a positive association between response (categorized as: NR, <VGPR; VGPR+CR) and progression after response assessment (*P* = 0.024; Fig. 2A). As far as previous exposure to bortezomib was concerned (8/24 patients, 33%), we observed 62% of ≥PR (five patients), with two patients (25%) with a VGPR (%Table 2), without difference in terms of percentage of responsive patients (*P* = 0.25) and PFS when compared to bortezomib-naive patients (median PFS 20.5 vs. 19.1 months, *P* = 0.87; Fig. 2B).

**Table 2:** Efficacy. Overall Response Rate of Patients Treated with B-MuD, and Response Rate According to Number of Previous Lines and Previous Bortezomib Exposure

Response	All patients % (*N*°)	According to previous line % (*N*°)				Previous Bor exposure% (*N*°)					
1–2	≥3	Yes	No
Drop-out	25% (6)	27% (4)	23% (2)	25% (2)	25% (4)
CR	8% (2)	7% (1)	11% (1)	0	13% (2)
VGPR	33% (8)	33% (5)	33% (3)	25% (2)	37% (6)
PR	21% (5)	13% (2)	33% (3)	37% (3)	13% (2)
SD	9% (2)	13% (2)	–	13% (1)	6% (1)
Progr	4% (1)	7% (1)	–	–	6% (1)

### Treatment exposure and adverse events

Patients received a median of 6 (range: 1–6) treatment cycles, with a median 93% of dose delivered. Dose modification occurred in nine patients (37.5%): bortezomib dose reduction without need for fotemustine modification occurred in five patients (21.5%) for emerging PN, four patients (16%) discontinued treatment (two for infections, 1 for grade IV sensory-motor PN, 1 for gastrointestinal bleeding). %Table 3 summarizes adverse events of any grade occurred during treatment, %Table 4 shows patient flow and toxicity according to dose delivered. Thrombocytopenia was the most common AE overall (83%, *n* = 20 patients). Grade 3–4 thrombocytopenia occurred in eleven patients (45%; four patients enrolled in the first cohort of six treated according to the original schedule, seven patients out of eighteen treated according to the amended bortezomib schedule). Need for platelets support occurred in two patients registering platelet values ≤10·10^9^/l (8%). Incidence of grade 3–4 neutropenia was 25% (six patients), with two patients (8%) with ANC ≤0.5·10^9^/l. Incidence of PN of any grade was 62.5% (15 patients). Focusing on grade 3–4 PN; the incidence of severe PN was 21% (five patients: one patient had severe disautonomic symptoms, four patients had sensory-motor neuropathy); disautonomic symptoms completely resolved by data cut-off, three out of four patients with grade 3–4 sensory-motor neuropathy experienced neurologic symptoms improvement, the remaining one had a partial improvement. Two of severe neurologic AE occurred in the first cohort of six patients treated according to the original twice weekly bortezomib schedule. Incidence of grade 3–4 extra-hematologic AE different from PN, was the following: infections 25% (six patients; three with febrile neutropenia, three with pneumonia), gastrointestinal symptoms 12% (two patients with severe nausea and diarrhea, one patients with gastrointestinal bleeding), metabolic alterations 8% (two patients with hyperglicemia). All those adverse events resolved or improved except one death reported as a consequence of NCI grade 4 infection (mycotic pneumonia) occurred in one patient treated with B-MuD as fifth-line therapy.

**Table 3:** Treatment Exposure and Adverse Events Reported During Therapy

	**N° of patients (%)**
Dose modification:	**9 (37%)**
Dose reduction	5 (21%)
-Fotemustine	2
-Bortezomib	5
-Dexamethasone	4
Treatment discontinuation	4 (16%)
	Total	Grade 1–2	Grade 3–4
Adverse events:			
Haematologic:	20 (83%)		
Neutropenia	8	2	6
Thrombocytopenia	20	9	11
Anemia	7	6	1
			
Infections:	13 (54%)		
Febrile neutropenia	3	–	3
Influenza A (H1N1)	1	1	–
Pneumonia	3	–	3
Upper respiratory tract	5	5	–
Urinary tract	1	1	–
Neuropathy:	15 (62.5%)		
Peripheral neuropathy	12	8	4
Dysautonomia	3	2	1
Constitutional (Fatigue)	2 (8%)	2	–
Gastrointestinal	9 (37.5%)		
Nausea	5	3	2
Diarrhea	3	1	2
Bleeding	1	–	1
Vascular			
Deep vein thrombosis	2 (8%)	2	–
Cardiac			
Atrial fibrillation	1 (4%)	1	–
Metabolic			
Fasting glucose value >250 mg/dl	2 (8%)	–	2

**Table 4:** Patient Flow and Toxicity According to Dose Delivered

Planned dose	N° of Patients	N° and % of discontinuation (reason for discontinuation)	N° and % of patient with grade 3–4 hematological toxicity	N° and % of patient with grade 3–4 non hematological toxicity
Fotemustine 80 mg/m^2^ + twice weekly Bor	6	2 (33%) (100% toxicity)	5 (83%)	6 (100%)
Fotemustine 80 mg/m^2^ + once weekly Bor	6	0	4 (66%)	2 (33%)
Fotemustine 100 mg/m^2^ + once weekly Bor	12	4 (33%) (50% toxicity—50% progression)	8 (66%)	6 (50%)

Bor = Bortezomib.

## Discussion

Despite the improvement of outcome observed in last decade, the majority of MM patients, ultimately relapsed 1,2. Relapsing patients, particularly patients in more advance stage, might benefit of therapeutic strategies including novel agents, such as the proteasone inhibitor bortezomib or IMiDs, as the backbone of two or more drug combination regimen. This phase I/II trial evaluated the efficacy and the safety profile of the three drug combination B-MuD in which bortezomib and dexamethasone were combined with fotemustine, a cytotoxic alkylating agent belonging to the nitrosureas family. This study followed the preliminary data regarding the efficacy of the single agent fotemustine observed in a small cohort of heavily pretreated MM patients 6.

The adoption of the longer 35-day schedule than the conventional 21-day schedule of fotemustine 5,6 combined with a once-weekly rather than the conventional twice weekly administration of bortezomib 24–26 allowed an equal cumulative dose delivered over the course of a longer period with a consequent significant improvement of the safety profile, especially in terms of lower incidence of haematological toxicity and neuropathy. In fact, despite the fact that thrombocytopenia was commonly observed (45% of patients), the adoption of the longer 35-day schedule, significantly reduced its incidence (38 vs. 80%). A further improvement in terms of neurological tolerability maybe obtained adopting subcutaneous bortezomib administration 27,28. The good tolerability of this regimen was also reflected by the high median percentage of the planned doses delivered (93%). Overall there were few patients (four patients, 17%) not completing the protocol due to toxicity, half enrolled in the first cohort of six treated according to the shorter and more intensive original schedule. In our study, a very small fraction of patient (4%) had renal failure, anyway fotemustine, differently from melphalan, would not require dose adjustment in presence of renal impairment.

The good safety profile of B-MuD regimen was associated with a significant efficacy. We observed a high proportion of patients (41%) reaching at least a VGPR (CR 8%), with a final ORR of 62%. These response rate were comparable to those obtained with three drug combinations such as bortezomib plus dexamethasone with the alkylating agent melphalan 7–9 or with the immunomodulating agent lenalidomide (RVD regimen) 29 and similar to those obtained with the four drug combination VMPT including thalidomide in addition to bortezomib, melphalan and prednisone 30.

Response occurred rapidly (median time to first response 36 days) and were long-lasting with a median PFS of 19.1 months (95% CI 11.9–22.2) overlapping data reported by other authors using similar three drug combinations 7,9,31–33. Outcome were even better if patients received B-MuD as second line therapy with a median PFS of 21.3 months. Response was significantly associated with outcome (*P* = 0.024) 16,34, with similar clinical benefit for patients reaching at least a partial response when compared to patients with good responses (median PFS 19.1 vs. 22.2 months in patients with PR and ≥VGPR respectively, *P* = 0.3).

As the comparable results observed in patients previous exposed versus naive to bortezomib, this study confirms the feasibility of bortezomib retreatment 35,36, particularly in combination with chemotherapy, reserving the use of different drugs such as next generation proteasome inhibitors or IMIDs in more advance disease phase.

Taking into account the good synergism with bortezomib and the good toxicity profile, fotemustine could represent a good alternative to alkylating agents in relapsed/refractory patients.
